# Comprehensive Review of FinFET Technology: History, Structure, Challenges, Innovations, and Emerging Sensing Applications

**DOI:** 10.3390/mi15101187

**Published:** 2024-09-25

**Authors:** Koosha Karimi, Ali Fardoost, Mehdi Javanmard

**Affiliations:** Department of Electrical and Computer Engineering, Rutgers, The State University of New Jersey, New Brunswick, NJ 08854, USA; koosha.karimi@rutgers.edu (K.K.); fardoost.ali@rutgers.edu (A.F.)

**Keywords:** short channel effect (SCE), FinFETs, multi-gate technology, high-k dielectrics, device performance metrics, biosensors, fabrication materials, Gas Sensing, pH Sensing, Ion Sensing, Temperature Sensing

## Abstract

The surge in demand for 3D MOSFETs, such as FinFETs, driven by recent technological advances, is explored in this review. FinFETs, positioned as promising alternatives to bulk CMOS, exhibit favorable electrostatic characteristics and offer power/performance benefits, scalability, and control over short-channel effects. Simulations provide insights into functionality and leakage, addressing off-current issues common in narrow band-gap materials within a CMOS-compatible process. Multiple structures have been introduced for FinFETs. Moreover, some studies on the fabrication of FinFETs using different materials have been discussed. Despite their potential, challenges like corner effects, quantum effects, width quantization, layout dependencies, and parasitics have been acknowledged. In the post-planar CMOS landscape, FinFETs show potential for scalability in nanoscale CMOS, which leads to novel structures for them. Finally, recent developments in FinFET-based sensors are discussed. In a general view, this comprehensive review delves into the intricacies of FinFET fabrication, exploring historical development, classifications, and cutting-edge ideas for the used materials and FinFET application, i.e., sensing.

## 1. Introduction

The electronics industry is undergoing a relentless trend toward miniaturization, and this transformation is especially notable in the realm of MOSFETs. Nevertheless, as these devices shrink to dimensions below 32 nm, a host of electric performance issues come to the forefront. These issues encompass the escalation of detrimental effects like Short Channel Effect (SCE), Gate Induced Drain Leakage (GIDL), diminished low power performance, gate direct tunneling leakage, Drain Induced Barrier Lowering (DIBL), subthreshold leakage current (Ioff) increase, and threshold voltage (Vth) shifts. In response to these challenges, Multi-Gate Field Effect Transistors (MuGFETs) have emerged as the most practical and effective alternatives for downsizing planar MOSFETs. This transition to multi-gate structures offers a powerful solution to overcome the limitations posed by shrinking device sizes while enhancing overall performance and reliability [1,2,3,4,5,6]. Some of these designs, like Dual Gate FinFET and Tri gate FinFET, benefit from thin Si-film, lightly doped channels, and double gates for better channel control [1].

Using MuGFET instead of planar MOSFETs leads to a better performance of the circuit, such as improving stability, higher I_on_/I_off_ ratio, decreased Short Channel Effect (SCE), low intrinsic gate capacitance, higher speed of operation, steep subthreshold slope [1,2,6], and improvement of consistency against random dopant fluctuation [5]. For example, a 16 nm/14 nm FinFET process could offer a 40–50% performance increase or a 50% power reduction compared with a 28 nm process [7].

FinFET is one of the MuGFETs that can be used as a substitute for the planar MOSFET. Of note is that the word “FinFET” comes from its visual shape, which is similar to a fish’s dorsal fin [6]. FinFETs marked the first significant architectural shift in transistor device history, introducing trigate control to extend gate-length scaling for several more generations [8,9]. That said, recently, in 2005, Ravariu introduced nothing on insulator (NOI) transistors that showed better performance in terms of subthreshold gate swing (SS) in comparison to FinFETs [10,11].

A more advanced version of MuGFETs, the gate-all-around FET (GAA-FET), surpasses FinFET and other sub-22 nm device architectures due to its superior gate coupling, which allows for more precise and accurate channel tuning. In GAA device architecture, Short Channel Effects are reduced compared with FinFETs at the same technology node [12]. Among the gate-all-around architectures investigated by the semiconductor industry, nanowires offer the best electrostatic control, while wider nanosheets provide higher “on” current and enhanced electrostatic control compared with FinFETs [13,14]. Gate-all-around (GAA) nanosheet field-effect transistors (FETs) have been widely adopted by the industry to continue logic scaling beyond the 5 nm technology node, surpassing FinFETs [15]. This represents only the second instance (following FinFET) in transistor device history where the industry has embraced a completely different architecture [16].

Furthermore, the aforementioned varieties of FinFETs, Double Gate FinFETs (DG FinFET) and Tri-Gate FinFETs are the most popular kinds of FinFETs that are used due to their excellent SC characteristics and smaller parasitic capacitance [1,3]. Also, their simple structure and simplicity in the fabrication process make them a perfect candidate for this purpose [5]. In comparison, Tri-Gate FinFETs have less fringing capacitance but are more complicated in the fabrication process [3]. However, it should be noted that there are still some challenges in fabricating FinFETs [2].

Currently, FinFETs are a focal point of research for numerous research groups, with ongoing exploration of their capabilities in a variety of applications. While substantial progress has been made, there is still a vast landscape of unexplored circuit implementations for FinFET technology [3]. Resolute scientists and engineers have harnessed the potential of various FinFET variants to create innovative FinFET-based sensors for diverse applications. This ongoing research and development holds the promise of unlocking even more potential applications and performance enhancements in the future.

## 2. History

### 2.1. Genesis of FinFETs

**Figure 1:**
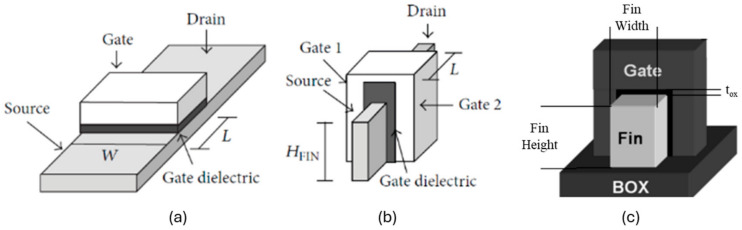
Structure Schematic of (**a**) planar MOSFET and (**b**) FinFET. (**c**) Demonstrating H_Fin_ as fin height and W_Fin_ as fin width [5,17].

Based on the prior records and documented history, the start of multi-gate transistors can be attributed to Hieda and colleagues in 1987 [1]. Their pioneering work revealed that these transistors, characterized by reduced body bias effects, exhibited superior switching performance when compared to conventional MOSFETs. Subsequently, in 1989, Hisamoto and his team introduced the first double-gate transistor, named DELTA [1,3,4,5]. This development garnered significant attention due to its marked reduction in the Short Channel Effect (SCE) phenomenon, setting it apart from traditional planar MOSFETs [1,5]. In Figure 1, we present a schematic illustrating the structural differences between planar MOSFETs and FinFETs for a clearer visual comparison.

### 2.2. What Is Fin?

The distinction between FinFETs and planar MOSFETs lies in their channel orientation, with FinFETs featuring vertical “fin” channels while planar MOSFETs employ horizontal channels. Figure 1c illustrates the fin height (HFin) and width (WFin). It is noteworthy that the fin height in FinFETs serves as the counterpart to the channel length in MOSFETs, a critical parameter that significantly impacts device performance and characteristics [1,3]. Increasing the number of fins in FinFETs results in higher charge density within the channels, translating to more precise gate control, thereby enhancing overall device performance [1,3]. However, it’s imperative to acknowledge that practical constraints in manufacturing and device performance necessitate the use of FinFETs with relatively smaller fin heights. This is because overly tall fins can push the device into an unstable operational regime, a phenomenon well-documented in the literature [1,3,4,5]. In engineering designs, standard practice is to maintain the fin height at a value lower than four times the fin thickness to ensure stable and reliable operation [5]. To determine the channel length of FinFETs, one can simply calculate it by multiplying the number of fins by the length of each individual fin, a straightforward relationship that plays a pivotal role in the fine-tuning of device parameters [4,5].

## 3. FinFET Structure Classification

FinFETs can be categorized into different aspects, which are as follows.

### 3.1. Based on Physical Structures

**Figure 2:**
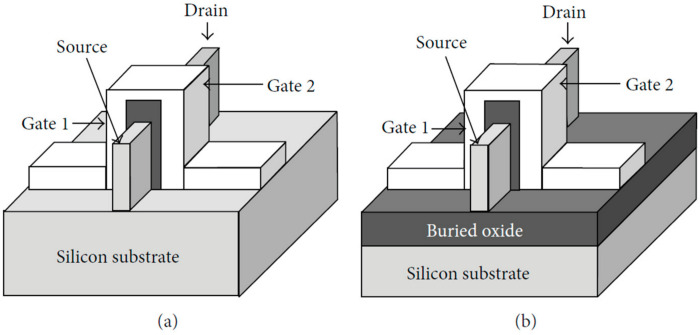
Structure schematic of (**a**) Bulk FinFET and (**b**) SOI FinFET [5].

FinFET technology is categorized into two main types: Bulk FinFETs and SOI (silicon on insulator) FinFETs, each characterized by distinct structural and operational features. In Bulk FinFETs, the individual fins share a common substrate, leading to their physical connection. On the other hand, SOI FinFETs are designed with physically isolated fins that do not come into direct contact [3,5]. For a visual reference, please consult Figure 2, which illustrates the structural disparity between these two FinFET types. Bulk FinFETs closely resemble the traditional planar MOSFET structure, making the transition from planar MOSFETs to Bulk FinFETs a relatively straightforward process. This structural similarity has led many companies to prefer Bulk FinFETs due to the ease of integration [5]. Nevertheless, the choice between Bulk FinFETs and SOI FinFETs remains a topic of debate among engineers, hinging on factors such as cost and performance considerations. The decision regarding which type to adopt depends on a complex interplay of these variables and specific design requirements [5].

### 3.2. Based on the Number of Terminals

**Figure 3:**
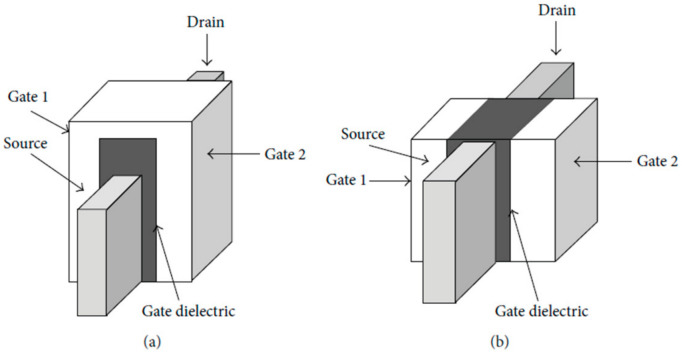
Structure schematic of (**a**) SG FinFET and (**b**) IG FinFET [5].

FinFETs can be categorized into two primary types based on their number of terminals: Short Gate (SG) FinFETs, with three terminals, and Independent Gate (IG) FinFETs, featuring four terminals. The key distinction between these two categories lies in their structural design. In SG FinFETs, two gates are shorted and physically connected to each other, while in IG FETs, the gates are physically isolated by a dielectric material [1,3,4,5]. Figure 3 provides a visual representation of the structural differences between these two types of FinFETs.

When we compare SG and IG FETs, a clear distinction emerges: SG FETs exhibit higher Ion and Ioff values. Conversely, IG FETs provide greater versatility in transistor control by allowing the application of varying voltages and signals to the gate terminal. However, this increased flexibility in IG FETs necessitates a more comprehensive approach to fabrication [1,3,5]. Furthermore, IG FETs yield a superior Ion/Ioff ratio due to their unique ability to adjust one gate’s voltage, either up or down, through the other gate. Consequently, IG FETs are better suited for applications in power management [1].

### 3.3. Based on Dielectric Thickness

**Figure 4:**
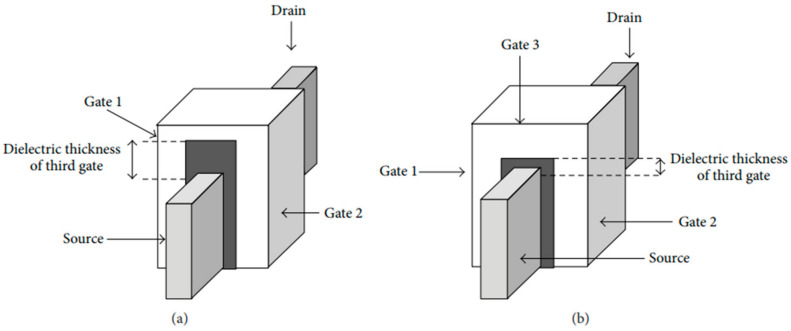
Schematic of (**a**) DG FinFET and (**b**) tri-gate FinFET [5].

Furthermore, FinFETs can also be classified based on their dielectric thickness into two main categories, as seen in Figure 4: double-gate (DG) FinFETs and tri-gate FinFETs. In DG FinFETs, a hard mask is employed atop the transistor structure, ensuring that the effective channel width is equal to two times the fin height (2n × Fin height). This type of FinFET is known as a split transistor [18]. In contrast, tri-gate FinFETs exhibit a unique feature. When the dielectric thickness is reduced, the effective channel width in tri-gate FinFETs becomes equivalent to the DG FinFET’s channel width plus the width of the fin (W_fin_). Consequently, the total channel width in tri-gate FinFETs is calculated as 2n × Finheight + W_Fin_. This design variation results in tri-gate FinFETs having a lower gate-to-source capacitance, offering distinct advantages in certain applications and better performance, while the electrostatic integrity remains strong as it has tri-gate in the device [1,19,20].

## 4. Various Materials Used in FinFETs

### 4.1. Materials Used in FinFET Fabrication

In a study, various types of materials were used and studied, including FinFET-based Dual KK-structure, InGaAs-on-Insulator FinFET, double-gate based n-FinFET using Hafnium oxide, SOI-FinFETs, MosFET (multi-gate), Deeply Scaled CMOS, FinFET, Selective Epitaxial Si Growth in FinFET, and Atomic Layer Deposition (ALD) in FinFET. A n-FinFET utilizing various gate materials, such as aluminum, molybdenum, and gold, was simulated [18].

A crucial role is played by the asymmetric drain extension dual-KK structure in the FinFET structure, optimizing parameters such as cutoff frequency (fT) and maximum oscillation frequency (fmax) [18]. Dual-kk is a type of spacer dielectric material used in the manufacturing of nanoscale devices such as FinFETs. It is a combination of two different high-k dielectric materials, one on the source side and the other on the drain side of the device. Efficiency improvement is observed in the asymmetric drain extension dual-KK structure compared with the dual-K structure. Another noteworthy material for FinFET is In-GaAs-on-Insulator, which is optimized based on the on/off trade-off, demonstrating record performance with an expanded gate length of up to 20 nm and a width of up to 10 nm [18,21,22]. This achievement is facilitated by a meticulous design of source/drain spaces and doped extensions to manage off-current migration [23]. The design and simulation of the double gate-based n-FinFET at 22 nm and 20 nm technology shows reduced leakage current through the use of Hafnium oxide. Hafnium oxide, known for its high-k dielectric constant as a gate dielectric, in combination with gold gate metal, exhibits a larger I_ON_/I_OFF_ ratio compared with aluminum [24]. Within the silicon-on-insulator (SOI) FinFET structure, fully depleted nMOS and pMOS FinFETs have been successfully demonstrated, featuring fin widths down to 5 nm and 65 nm tall fins [18]. The comparison between the mentioned materials is demonstrated in Table 1.

### 4.2. Gate Dielectric Material

The semiconductor industry aims to incorporate high-k gate dielectrics in double-gate transistors to reduce leakage current while balancing energy consumption. The multi-gate device exhibits the potential for expansion beyond bulk planar CMOS with high-k dielectric materials, leveraging strong electrostatic control over the channel. By varying the gate-lap length and incorporating high-k gate insulating material into the FinFET device structure at the 10 nm technology node, improvements are observed in the electrical performance current ratio (Ion/Ioff) and DIBL. Notably, HfO_2_ gate oxide material demonstrates a high drain current, and a decrease in the k value of the gate-oxide material directly impacts the current performance [25]. Deploying high-k dielectric materials in the narrow channel length of FinFET devices is essential for enhancing efficiency and minimizing SCEs. The industry’s primary focus is on designing FinFETs using innovative dielectric materials, including Al_2_O_3_, SiO_2_, HfO_2_, Si_3_N_4_, ZrO_2_, TiO_2_, Y_2_O_3_, Ta_2_O_5_, and LaZrO_2_, which reduce gate leakage current. Despite direct tunneling occurring due to a reduction in the gate oxide layer thickness, the utilization of high-k dielectric materials addresses challenges and reduces power consumption [26]. DG FinFETs benefit from gate dielectrics, which restrict current passage across the gate, providing advantages such as low leakage current, larger drain current, and improved electrical properties with higher gate dielectric materials [19].

The impact of using different gate dielectrics is explored on three specific electrical characteristics of FinFETs. Looking at Vth fluctuations, TiO_2_ exhibits a greater Vth value, leading to improved performance [26]. Moreover, sub-threshold swing values reveal that TiO_2_ has a lower SS due to its high dielectric and lower leakage current [26]. In addition, DIBL values also show TiO_2_’s superiority in current flow control over the gate channel compared to different dielectric gate materials by a 96% drop [26]. The multi-gate design, coupled with high-k dielectric materials, enhances speed performance in electric circuits while mitigating device difficulties. To reduce leakage current in short-channel devices, high-k-value dielectric materials like TiO_2_ are necessary. Generally, a high dielectric constant results in a maximum Ion/Ioff ratio, which is crucial for applications such as loudspeakers and electronic signals [19,27,28].

LaZrO_2_, as a cutting-edge material with a high dielectric constant and broad energy band-gap, has been employed in 14 nm FinFETs, per the International Technology Roadmap for Semiconductors (ITRS), showing improved Ion/Ioff ratio and decreased Ioff compared with SiO_2_ [29,30]. The electrostatic potential of the device rises toward the drain terminal with high-k dielectric gate material, improving gate capacitance and minimizing DIBL [31]. In high-k gate dielectrics like LaZrO_2_, the on-current increases (by 2.7), off-current decreases (by 101), and SS (by 10%) and DIBL (by 76%) are lowered compared to SiO_2_ [19]. TiO_2_, on the other hand, as a gate dielectric material, improves threshold voltage and reduces short-channel effects, while HfO_2_ acts as a durable high-k dielectric oxide material with significantly lower leakage current than SiO_2_. These high-k dielectric materials offer a higher dielectric constant than traditional SiO_2_, resulting in lower current leakage and improved thermal stability [19]. Table 2 compares the impact of SiO_2_ and LaZrO_2_ on an n-FinFET device efficiency.

### 4.3. Channel Material

Some studies have been conducted regarding different channel materials of FinFETs and their performances. In a study, scientists designed and simulated an SOI FinFET device with different channel materials, including Si, GaAs, SiGe, and SiC3C. The FinFET device with the material silicon in its channel showed the highest I_ON_ (5.03 × 10^−6^ A), while the device designed with GaAs had a minimum subthreshold swing (SS) of 58 mVdec. Moreover, devices with SiC3C showed the maximum ratio of I_ON_/I_OFF_ (1.90 × 10^11^) and minimum I_OFF_ (7.00 × 10^−19^ A) [32].

Also, it has been investigated that in order to have better DIBL qualities, we need to use GaN and GaAs for channel material. Regarding threshold voltage roll-off characteristics, GaN showed the best performance among the other materials. The poorest material for the channel would be GaSb, which performed very bad short channel effect characteristics [33,34]. Moreover, using CNT and Graphene can improve the ratio of I_ON_/I_OFF_, improve the speed, and reduce energy consumption [4,35].

## 5. FinFET Challenges

The impact of transitioning from planar to FinFET technology is explored in different areas, including general difficulties, like corner effects, parasitic capacitance, etc. and fabrication intricacies, like channel doping concentration, silicon surface variations, and fin oxidation, emphasizing the need for careful consideration and solutions in these areas. Solution to each of them is also provided [7].

### 5.1. Phenomena in FinFET

#### 5.1.1. Parasitic Capacitance

**Figure 5:**
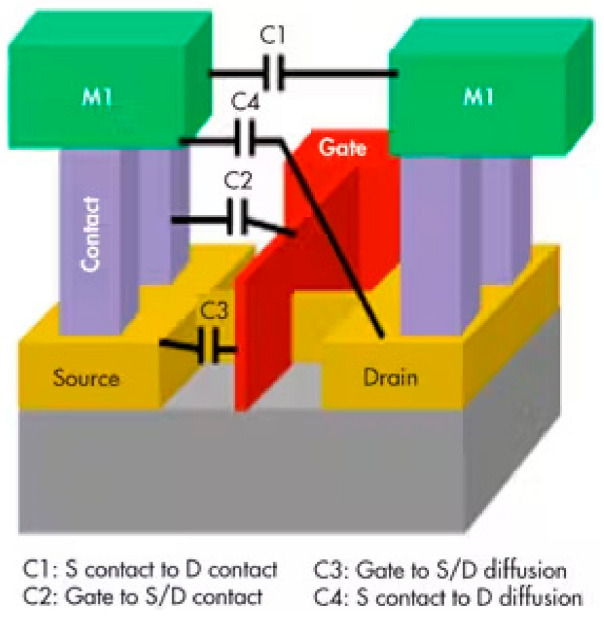
Parasitic capacitances of a FinFET [7].

FinFET devices exhibit a higher incidence of parasitic capacitances compared with their planar MOSFET counterparts, primarily due to the increased overlap region between the front and back gates. To mitigate these parasitic capacitances, an effective approach involves elevating the fin height while simultaneously reducing the fin pitch [1,3,7,36,37]. Figure 5 shows these capacitances.

#### 5.1.2. Corner Effects

**Figure 6:**
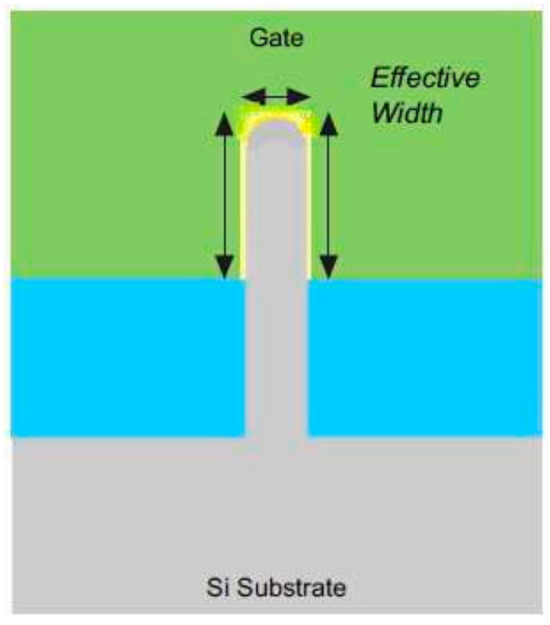
Cross-sectional view of a Tri-FinFET with curved fin corners [7].

Decreasing the fin-width is effective in reducing short-channel effects; however, it comes with the trade-off of potential performance degradation in FinFET. This degradation is attributed to the increase in parasitic drain/source resistance, resulting in a reduction of drive current and trans-conductance of the device. The higher concentration of subthreshold leakage current at the corners of the fin due to the aforementioned events is called the “corner effect” [38]. Furthermore, the smaller fin width impedes the easy flow of heat, leading to an increase in device temperature. The solution to this problem is that the FinFET production features a more curved and rounded profile, as seen in Figure 6 [7].

#### 5.1.3. Quantum Effects

If the FinFET is excessively thick, the electrostatic influence of the gate on the fin’s sides and top weakens, causing the fin body to mimic a bulk substrate and forfeit the advantages of the FinFET topology. Conversely, in the case of an extremely thin FinFET, the density of available electron (or hole) states diminishes. While there is typically an abundance of “free states” for energetic electrons/holes at the band edges, the quantum effect in very thin fins reduces the density of available states at the band edge. Consequently, electrons/holes necessitate more energy to occupy states higher than the band edge and be liberated to conduct device current [7].

#### 5.1.4. Crystal Orientation for Fin Surface

Surface orientation and current flow direction play crucial roles in determining the mobility of electrons and holes. The (100) orientation stands out with the highest surface mobility for electrons, contrasting with the lowest observed in the (110) orientation. On the other hand, holes showcase their peak mobility in the (110) orientation and the least in the (100) orientation. The (111) surface orientation introduces a middle ground, with the mobility of electrons and holes lying between the (100) and (110) surface orientations. These fluctuations in mobility are a result of surface scattering phenomena [1,3,39,40].

#### 5.1.5. Threshold Voltage Adjustment

As the FINFET channel achieves full depletion, the adjustment of the threshold voltage is confined to the gate work function (WF) dictated by the metal electrode. A moderate threshold voltage (V_T_) can be achieved by employing a mid-gap metal like TiN. However, to attain a low V_T_, the work function requires the implementation of additional cap layers (such as La_2_O_3_ or Dy_2_O_3_ for NMOS and Al_2_O_3_ for PMOS) positioned between the high-k dielectric and the metal electrode. Given the critical importance of the thickness of these layers, their deposition necessitates Atomic Layer Deposition (ALD) processes to ensure a conformal gate stack around the fin [2,40,41].

### 5.2. Fabrication Drawbacks

#### 5.2.1. Doping Concentration

While FinFET channels do not necessitate doping, light doping is often introduced for the purpose of monitoring leakage current and threshold voltage. However, this practice can lead to elevated series resistance in the source and drain regions. Also, the necessity for the implementation of multiple work functions is another consequence of this problem [7]. To address this issue, the incorporation of epitaxial growth within the fabrication process becomes imperative [1,3,42].

#### 5.2.2. Integration Challenges

**Figure 7:**
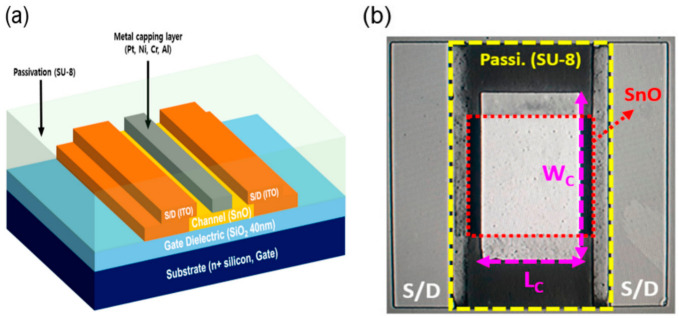
(**a**) 3D view; (**b**) top view optical image of a transistor with a metal capping layer [44].

In the fabrication process, creating the patterns for the fins and gates in FinFETs demands a significantly more intricate level of process control compared with planar MOSFETs. For instance, ensuring a stable threshold voltage in gate lengths below 20 nm necessitates achieving a fin width as narrow as 10 nm, with a fin width of only 1 nm, a challenging task. Additionally, due to the elevated topography and the presence of a high-k dielectric in the gate stack, the introduction of a cap layer becomes essential for fine-tuning the threshold voltage, as seen in Figure 7. Consequently, gate patterning emerges as a formidable challenge in this context [2,3,43].

Double patterning, involving the utilization of two masks to print alternating features, becomes essential at 20 nm and below to ensure accurate printing of features using existing lithography equipment. Moreover, layout-dependent effects (LDE) arise when features in the layout positioned close to a cell or device influence its timing and power [7].

#### 5.2.3. Junction of Multiple FinFETs

The significant series resistance in FinFET devices profoundly undermines their operational speed, a predicament attributed to the thin body and the intricate nature of forming a 3D structure. Although conventional doping techniques can be employed on the sidewalls of the fins with minor implantation angles, this approach often leads to undesirable backside scattering. Conversely, the use of heavy ion implantation can result in the complete amorphization of the fins, impairing device integrity [2,45,46].

To tackle this issue, several alternative doping methods have been introduced, including Plasma Doping and Vapor Phase Deposition, offering more precise and controlled approaches to address the series resistance challenge [2,47].

#### 5.2.4. Fin Surface Considerations

The surface of fins (which is typically made of silicon) exhibits variations from the bulk, leading to noticeable Si loss during the standard pre-gate-oxide cleaning process. Consequently, wet cleans are fine-tuned with lower concentrations and temperatures. Additionally, the oxidation rate of fins is accelerated at the corners and tips [7].

## 6. Recent Breakthroughs in FinFET Technology

### 6.1. Manufacturing a 10 nm Gate Length FinFET

Here, the comprehensive exploration of the design, fabrication, performance, and integration aspects of a double-gate FinFET is discussed. The authors report the fabrication of the “first ever” double-gate FinFETs with a 10 nm physical gate length and a 12 nm fin width, claiming them to be the smallest double-gate transistors ever fabricated (at the time of the publication, in 2002). Fabricated on bonded SOI wafers through a modified planar CMOS process, the FinFETs employ dual-doped (n+/p+) poly-Si gates, utilizing ion implantations and subsequent RTA activation. A pattern reduction technique achieves sub-10 nm dimensions for both fin width and gate length. The gate insulator is a nitride oxide with a 17 Å physical thickness. The conducting channels form on the (110) crystal-oriented vertical sidewalls of the silicon fin, differing from the conventional (100) orientation in planar CMOS devices. It was seen that for a constant fin width when increasing the channel length from 10 nm to 100 nm, both n-FinFET and p-FinFET demonstrate a decrease in both DIBL and subthreshold swing. However, almost always, p-FinFET has a higher DIBL and SS than n-FinFET with the same channel length. So, n-FinFET usually operates better with lower short-channel effects than p-FinFET. For example, at 20 nm channel length, p-FinFET has a DIBL of 105 mVV and SS of 90 mVdec. On the other hand, the n-FinFET has a DIBL of 51 mVV and SS of 72 mVdec [48].

### 6.2. Ground Plane FinFET

**Figure 8:**
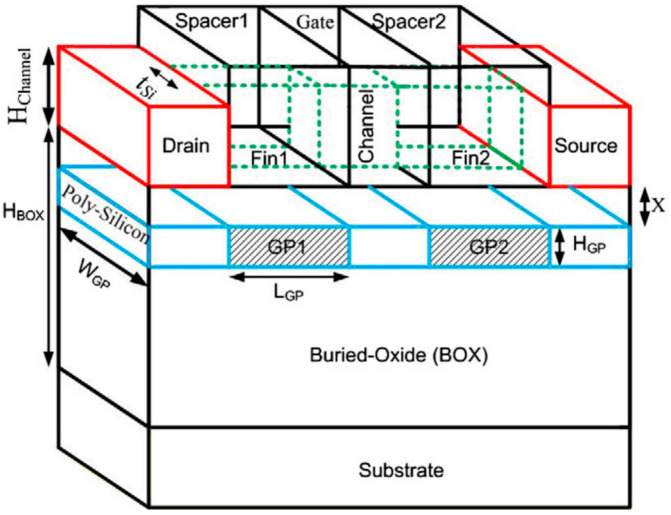
Three-dimensional view of the GP-FinFET structure schematic [53].

In another study, a FinFET structure that utilizes the ground plane concept was proposed and theoretically investigated. The ground plane aims to reduce the coupling of the electric field between the source and drain, thereby minimizing drain-induced barrier lowering (DIBL). The proposed structure, known as ground plane FinFET (GP-FinFET), introduces two ground planes under the source and drain regions [49,50,51,52]. To evaluate its performance, certain device characteristics were compared to silicon on insulator-FinFET (SOI-FinFET) and Bulk-FinFET structures. Unlike the conventional Bulk-FinFET, the GP-FinFET incorporates a buried ground plane and a BOX layer. The structure employs a tri-gate design and short-fin length, where corner effects may influence the electrostatic potential profile [49]. To mitigate this, tall spacers were employed [53]. Figure 8 demonstrates the schematic of the proposed structure.

Under constant gate overdrive voltage, GP-FinFET exhibits a larger drain current at high drain voltages compared with the other two structures. The results show a reduced subthreshold slope, leading to a significant reduction in leakage current and a higher ON/OFF current ratio for the proposed structure. As anticipated, the ground planes effectively reduce the DIBL effect and increase the threshold voltage. The read static noise margins (SNMs) are comparable among SOI-FinFET, Bulk-FinFET, and GP-FinFET structures, but GP-FinFET demonstrates the minimum standby power due to a higher threshold voltage and lower DIBL effect. Additionally, when employing the same gate work function, the read current of GP-FinFET is smaller than that of the other two structures incorporated in SRAM cells. The improvements become more pronounced as the channel length decreases [53].

### 6.3. Bottom Spacer FinFET

**Figure 9:**
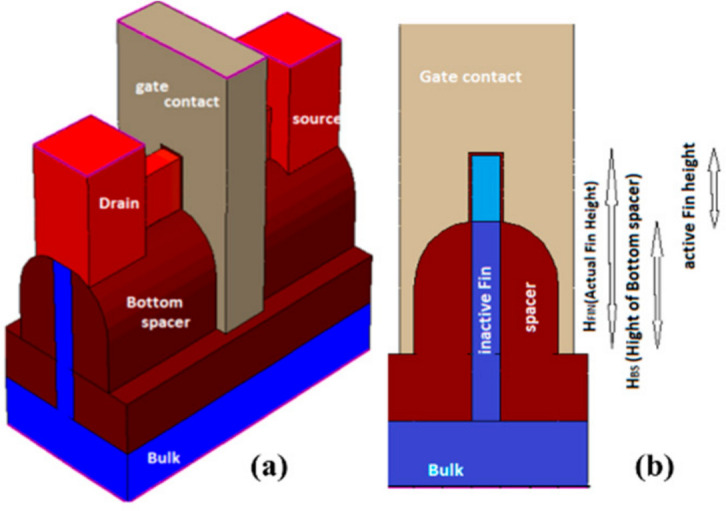
(**a**) 3D view and (**b**) side view of the structure of bottom spacer FinFET [55]. As can be seen, the active region is reduced after adding the bottom spacer.

The concept of reducing the active fin height to reduce power consumption and improve the short channel effect and self-heating performance was first used in 2010. A novel bottom spacer (BS) FinFET structure, which solves the problems caused by the width quantization effect and does not require any additional fabrication process except the standard FinFET process, has been proposed. The BS FinFET incorporates an added region between the fins, referred to as the BS, which ensures the insulation of the inactive fin area from gate contact. Consequently, this modification has led to notable improvements in various electrical parameters, including off-state current (I_off_), on-state current (I_on_), I_on_/I_off_ ratio, subthreshold slope, drain-induced barrier lowering (DIBL), and threshold voltage roll-off [54]. Figure 9 shows the structure of the BS FinFET with 22 nm channel length, 60 nm fin height, and 10 nm fin width.

### 6.4. Negative Capacitance FinFET with Ferroelectric Spacer

**Figure 10:**
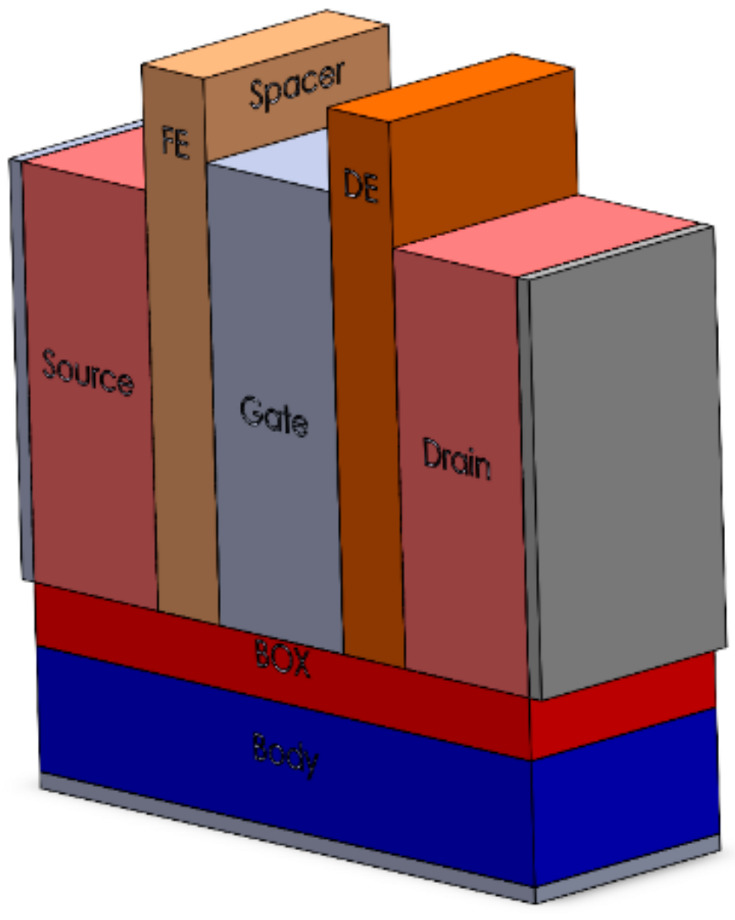
Three-dimensional view of the most effective structure of Negative Capacitance FinFET with Ferroelectric Spacer (Adapted from [56]).

Furthermore, in 2021, a novel concept was introduced in the form of the Negative Capacitance FinFET with Ferroelectric Spacer (NCFS). This concept emerged from the intent to amplify the electric field and charge in the extension region. Unlike traditional designs that utilize dielectric materials in the gate oxide stack, the NCFS employs ferroelectric materials. The utilization of ferroelectric materials facilitates polarization, leading to enhancements in both the on-state current (Ion) and the subthreshold slope. Four possible configurations have been explored, and among them, the structure in Figure 10 was the most efficient one (ferroelectric material between gate and source and dielectric material between gate and drain) [56].

### 6.5. Top-Bottom Gated FinFET

**Figure 11:**
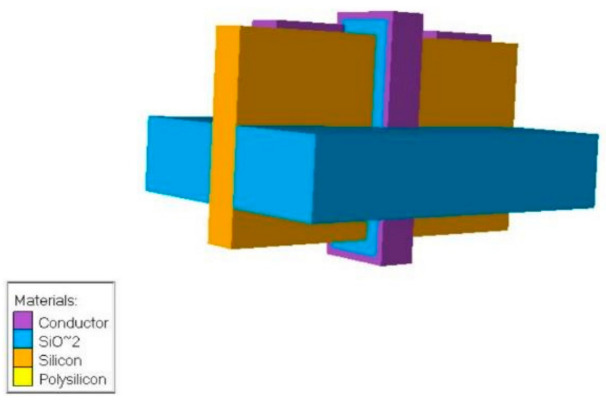
Three-dimensional schematic of Top-Bottom Gated FinFET [58].

In another study, a significant modification was implemented in the FinFET structure, which involved the incorporation of gates on both sides of the substrate. In this design, known as the Top-Bottom Gated (TBG) FinFET, which is demonstrated in Figure 11, the gate is strategically wrapped around the channel, with fins extending on both sides of the substrate. This distinctive configuration enhances the switching ratio and augments the control exerted by the gate over the channel. The TBG FinFET also exhibits superior performance in terms of higher drain current and transconductance parameters. Moreover, there is a noticeable improvement in the subthreshold swing in this modified structure [57].

## 7. Sensing Applications of FinFET

As mentioned in the previous sections, FinFETs have emerged as a critical component in modern nanoelectronics, offering superior performance in terms of speed, power efficiency, and scalability compared with traditional planar FETs. Their unique 3D structure and enhanced electrostatic control over the channel make them highly sensitive to changes in the environment, enabling their use in a wide range of sensing applications. FinFET-based sensors have shown remarkable potential in detecting various biological, chemical, and physical parameters with high accuracy and sensitivity. This section explores the diverse sensing applications of FinFET devices, highlighting their capabilities, advantages, and the emerging trends in their development.

### 7.1. Biosensors

FinFET devices, featuring a unique three-dimensional transistor structure, are promising for biosensing applications, transforming medical diagnostics. Their exceptional electrostatic control and reduced leakage current make FinFETs highly sensitive to small changes in electrical signals, which is crucial for biosensors and enables accurate detection of biomolecules like DNA and proteins. The low power consumption of FinFETs enhances their suitability for portable biosensing devices, while their miniaturized size allows the integration of multiple sensors on a single chip for the simultaneous detection of various analytes. These FinFET-based biosensing technologies show great promise for rapid, sensitive, and cost-effective point-of-care diagnostics, advancing personalized medicine and healthcare outcomes [59]. Another advantage of using FET-based biosensors is that they can be used in wet and dry environments [60]. In comparison to other semiconductor devices, FinFETs offer a larger surface-to-volume ratio that makes it a good choice in biosensors [61]. While FinFET devices have shown considerable promise in biosensing applications, there is still much work to be done to fully realize their potential and address existing challenges. In this section, we will briefly review some developments of FinFETs in biosensing applications.

#### 7.1.1. Label-Free Biosensing Using a Negative Capacitance FinFET

**Figure 12:**
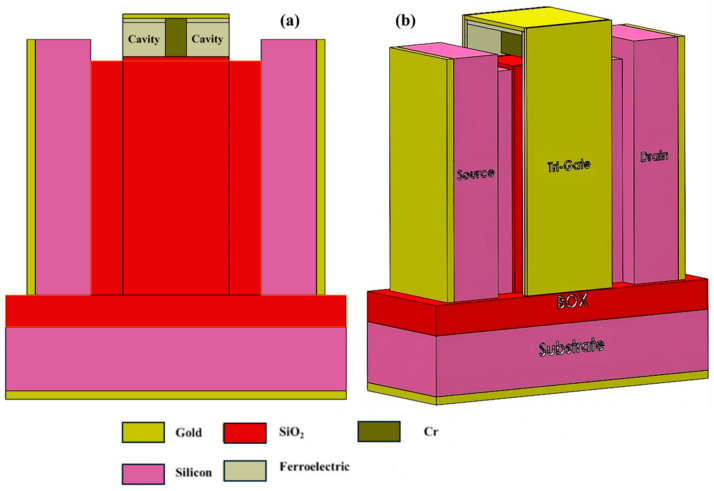
Depicting the proposed RSD NCFinFET schematic in (**a**) 2D cross-sectional view and (**b**) 3D view, showcasing diverse materials, layers, and the sensor electrode. The accessible cavity region for biomolecules, considering the channel orientation, is visible in the cross-sectional view (Adapted from [62]).

This study introduces a dielectric modulated (DM) negative capacitance (NC) FinFET biosensor for efficient label-free biomolecular detection. The NC effect enhances sensitivity and rapid response, and a raised source-drain (RSD) architecture increases sensitivity and selectivity due to more cavity space for biomolecules [62]. The schematic of this biosensor is included in Figure 12.

Operating on varying oxide capacitance, the biosensor utilizes a ferroelectric material for voltage amplification, resulting in increased current driving capacity, higher current ratio (CR), and lower subthreshold slope (SS) compared with conventional FET-based biosensors [63]. The proposed DM biosensor features a nanogap between passivated SiO_2_ and HfO_2_ layers, modulating the dielectric constant in the cavity [64]. Biomolecules influence electrical behavior, affecting effective oxide thickness, surface potential, and key electrical parameters. Among them, biomolecules with higher K-values increase threshold voltage and reduce OFF-state current. The presence of biomolecules in the cavity also reduces maximum drain current and output conductance and increases output resistance and intrinsic gain [63].

#### 7.1.2. Sub-20 nm GaAs Junctionless FinFET Biosensor

**Figure 13:**
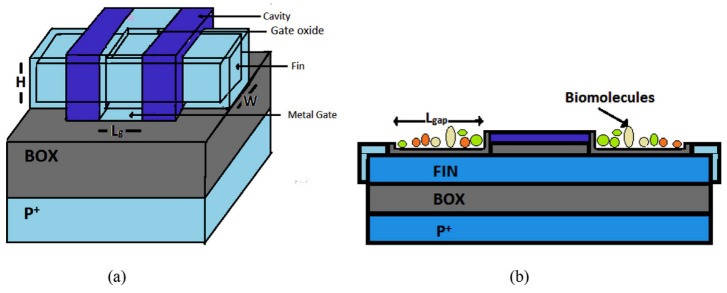
(**a**) 3D schematic and (**b**) side view of the sub-20 nm GaAs junctionless FinFET biosensor [58].

In another work, a dielectric-modulated GaAs junctionless FinFET as a sub-20 regime biological sensor, utilizing HfO_2_ (ᴋ = 25) to enhance the switching ratio threefold, was introduced. As seen in Figure 13, the biosensor features a nano-cavity (18 nm) for biomolecule immobilization, resulting in an 80% sensitivity improvement for biomolecule detection [58].

The study explored electrical characteristics and revealed distinct current changes for different biomolecules. Junctionless devices operate with the gate as a gated resistor, making them promising for biomolecule detection [61]. The proposed GaAs junctionless FinFET biosensor, with specific parameters, exhibits increased on-current, switching ratio, and sensitivity in the presence of protein biomolecules. The gap between the conduction band and the Fermi level widens as the dielectric in the cavity region increases when a biomolecule is present. Moreover, in the presence of protein within the cavity, a slight elevation in potential occurs along the channel region, resulting in an upsurge in the drain current. A decrease in the nano-cavity gap diminishes the switching ratio, indicating reduced biomolecule impact on device performance [58].

#### 7.1.3. Sub-40 nm Dielectric Based FinFET Biosensors

Here, we discuss some other works that addressed the use of change in dielectric constant in response to biomolecules’ presence. A label-free FinFET-based device has been developed and simulated and is used to detect neutral biomolecules such as keratin, bacteriophage, biotin, etc. The parameter that was used to sense these biomolecules is their dielectric constant. To see the sensor’s efficiency, a parameter called “sensitivity” was defined [65]. The proposed device’s distinct electrical features, including potential, switching ratio, on-current, and subthreshold slope, were examined for biomolecule detection. A comparative analysis of these characteristics was conducted, specifically with the presence of air within the cavity, to discern variations associated with different biomolecules. With the escalation of the dielectric constant within the cavity due to biomolecules, there is a corresponding rise in the parasitic capacitances of FinFET. Consequently, the drain current, switching ratio, and finally sensitivity increase with the increase of the dielectric constant [66]. In another similar study, a raised source-drain (RSD) FinFET was developed and employed to sense biomolecules. The concept of sensing biomolecules also differs in their dielectric constant [61].

### 7.2. Chemical Sensors

#### 7.2.1. Gas Sensing

In a study, Gandhi et al. evaluated a Junctionless (JL) FinFET-based hydrogen gas sensor and investigated the key challenges, such as self-heating effects (SHEs) and process variations like metal grain size and random dopant fluctuations. They presented a novel analysis of how these factors impact the sensor’s reliability, performance, and aging. The study shows that SHE can modulate sensor characteristics, reducing the impact on the ON current and lattice temperature during H₂ detection [67]. In another H_2_ sensing-based FinFET, the same scholars present a study on the junctionless negative capacitance FinFET (JLNC FinFET) for hydrogen (H₂) gas sensing. This work introduces a novel approach by integrating a ferroelectric (FE) layer into the FinFET structure, enhancing sensitivity through internal voltage amplification due to negative capacitance (NC). The analysis highlights that the JLNC FinFET shows improved sensitivity over traditional junctionless FinFETs, particularly under varying hydrogen gas pressures and temperatures [68].

Another research by Seghal et al. introduced a junctionless FinFET and Inverted Mode FinFET as phosphine gas (PH_3_) sensors. The analysis focused on various sensing parameters, like threshold voltage, drain current, and transconductance. The study found that junctionless FinFETs offer better sensitivity for parameters like threshold voltage and transconductance, whereas Inverted Mode FinFETs show superior performance in terms of ON/OFF current ratios, making each device more suitable depending on the specific sensing parameter being prioritized [69].

#### 7.2.2. pH Sensing

Rani et al. discussed the application of high aspect-ratio FinFETs as pH sensors, focusing on the novel integration of chemical functionalization with Aminopropyltriethoxysilane (APTES). The key innovation of this research lies in the use of FinFETs with a high height-to-width aspect ratio, which significantly enhances the signal-to-noise ratio and linearity of the pH sensing compared with traditional nanowire sensors. By functionalizing the SiO_2_ dielectric layer with APTES, the researchers were able to improve the sensitivity and linearity of the FinFETs’ pH response. The work also presented a novel method for vapor-phase APTES salinization using a home-built setup, offering a reliable and accessible approach for developing bio-FETs with potential applications in biosensing [70].

In another study, Alharbi et al. focused on the pH sensing performance of FinFETs by analyzing the effects of liquid gate and back gate capacitive coupling through 3D TCAD simulations. The novel contribution of this paper lies in demonstrating how the capacitive coupling ratio between the liquid gate and back gate can be tuned to amplify pH sensitivity beyond the Nernst limit, achieving a high sensitivity of 1.3 VpH. The study highlights that by adjusting the thickness of the buried oxide layer, the pH sensitivity can be fine-tuned, with the coupling ratio playing a critical role. Additionally, the work emphasizes that dual-gate operation enhances the signal-to-noise ratio (SNR), making the FinFETs highly suitable for pH sensing applications. The simulations show that the optimized FinFET structure provides superior sensitivity, linearity, and SNR compared with traditional planar structures, making it a promising candidate for advanced biosensing technologies [71].

#### 7.2.3. Ion Sensing

**Figure 14:**
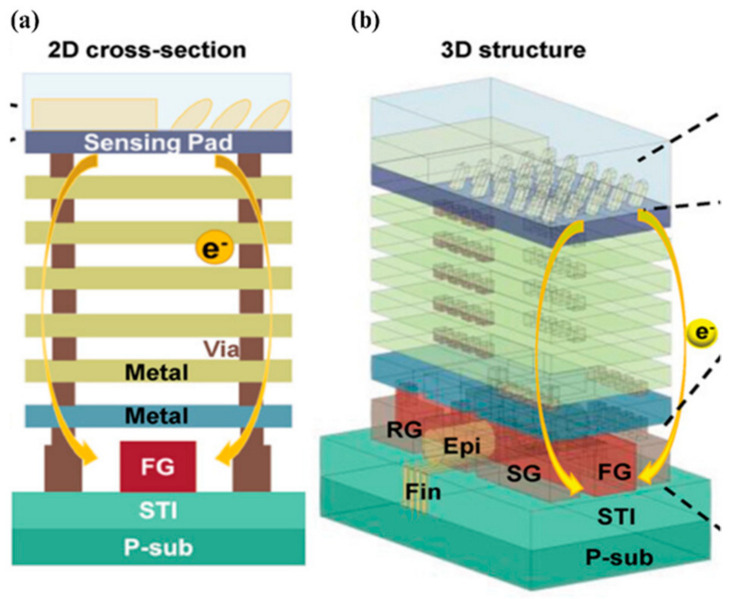
(**a**) 2D cross-section and (**b**) 3D structure of the ion-sensitive floating gate field effect transistor (ISFGFinFET). The FGFET is connected to the sensing pad (SP) by metal layers, and the sensing film is deposited on the SP [72].

Shen et al. unveiled a multifunctional ion-sensitive floating gate FinFET (ISFGFinFET) designed to detect hydrogen and sodium ions. The key innovation is the incorporation of a three-dimensional nanoseaweed structure fabricated via glancing angle deposition (GLAD), which significantly enhances the sensor’s surface area and, consequently, its sensitivity. The device demonstrates a high sensitivity of 266 mVpH for hydrogen ion detection and 432.7 mV per pNa for sodium ions. Additionally, the ISFGFinFET can detect multiple ions simultaneously, making it highly versatile for biomedical applications. The study also integrated a sensor array with 3 × 3 pixels and simulated a 16 × 16 array, paving the way for advanced ion sensing and spatio-temporal ion distribution analysis. This work offers a significant advancement in the design and functionality of ion sensors, particularly through the use of GLAD technology and the combination of different sensing materials like Al2O3 for enhanced performance [72]. Figure 14 demonstrates the schematic of the ISFGFinFET.

### 7.3. Physical Sensors

#### Temperature Sensing

Singh et al. reveal the innovative use of bulk FinFETs, fabricated using a 12 nm CMOS technology node, for Quantum Dot (QD)-based thermometry in cryogenic applications. This approach leverages the Coulomb blockade regime of FinFETs to develop high-speed, reliable temperature sensors that are operational within the 10.7 K to 40 K range, addressing the challenges of on-chip heating in Quantum Processing Units (QPUs). A novel aspect of this work is the integration of machine learning (ML) models to simplify temperature estimation, which traditionally relies on complex Fermi-function-based equations. By replacing these with linear models, the paper demonstrates significant reductions in training time and computational complexity, enabling real-time thermal monitoring [73].

## 8. Conclusions

In this paper, we reviewed the history, classifications, challenges, materials, and novel ideas of FinFET devices, which are promising candidates for the future of nanoscale CMOS technology. We discussed the advantages of FinFETs over planar MOSFETs in terms of electrostatic control, power consumption, scalability, and performance. We also explored the various types of FinFETs based on their physical structures, number of terminals, and dielectric thickness. Moreover, we examined the use of different materials for the fin, the gate, and the channel of FinFETs, as well as their impact on the device characteristics and parameters. Furthermore, we highlighted some of the fabrication challenges and drawbacks of FinFETs, such as doping concentration, integration issues, corner effects, and parasitic capacitances. Additionally, we presented some of the novel structures and concepts that have been proposed to overcome these challenges, such as ground plane FinFET, bottom spacer FinFET, and negative capacitance FinFET. Furthermore, FinFETs have shown potential for novel applications, such as various sensing applications, by exploiting their unique 3D structure and enhanced electrostatic control over the channel. In conclusion, FinFETs are a versatile and powerful technology that can enable future innovations in nanoscale CMOS and beyond.

## Figures and Tables

**Table 1:** Improvements made by using each material in FinFET fabrication [18].

Material Used in FinFET	Improvement
FinFET based dual KK-structure	(1) g_m_ ~ 9.09%
	(2) g_ds_ ~ 13.04%
	(3) Ft ~ 12.91%
	(4) Maximum oscillating frequency
InGaAs-on-Insulator FinFET	Improvement in the gate and source of drain length
Double Gate based n-FinFET using HfO_2_	Leakage current reduces
SOI-FinFETs	Fin width improvement
MosFET (Multi gate), Deeply Scaled CMOS, FinFET	Short channel effects and Leakage Issues are obtained. Improvement in chip area and high performance
Selective Epitaxial Si Growth in FinFET	Repairs the fin outer surface
Atomic Layer Deposition (ALD) in FinFET	Improvement in threshold voltage (Vt)

**Table 2:** The impact of SiO_2_ and LaZrO_2_ gate dielectric material usage in n-FinFET device efficiency [31].

Parameters (V_d_ = 0.75 V, V_g_ = 0.75 V)	(LaZrO2) Gate dielectric material k = 40	(SiO2) Gate dielectric material k = 3.9
I_ON_ (A)	4.95 × 10^−5^	1.78 × 10^−5^
I_OFF_ (A)	3.61 × 10^−14^	5.02 × 10^−13^
I_ON_/I_OFF_	1.37 × 10^9^	3.50 × 10^7^
V_t_ (V)	0.253	0.207
SS (mV/dec)	60.3	67.02
DIBL (mV/V)	10.1	43

## Data Availability

No new data were created or analyzed in this study. Data sharing is not applicable to this article.
